# Anti-Inflammatory Effect of ETAS®50 by Inhibiting Nuclear Factor-*κ*B p65 Nuclear Import in Ultraviolet-B-Irradiated Normal Human Dermal Fibroblasts

**DOI:** 10.1155/2018/5072986

**Published:** 2018-06-03

**Authors:** Ken Shirato, Tomoko Koda, Jun Takanari, Takuya Sakurai, Junetsu Ogasawara, Kazuhiko Imaizumi, Hideki Ohno, Takako Kizaki

**Affiliations:** ^1^Department of Molecular Predictive Medicine and Sport Science, Kyorin University School of Medicine, 6-20-2 Shinkawa, Mitaka, Tokyo 181-8611, Japan; ^2^Faculty of Nursing, Tokyo Healthcare University, 2-5-1 Higashigaoka, Meguro, Tokyo 152-8558, Japan; ^3^Amino Up Chemical Co. Ltd., 363-32 Shinei, Kiyota, Sapporo, Hokkaido 004-0839, Japan; ^4^Department of Health Science, Asahikawa Medical University, 2-1-1-1 Midorigaoka-Higashi, Asahikawa, Hokkaido 078-8510, Japan; ^5^Faculty of Human Sciences, Waseda University, 2-579-15 Mikajima, Tokorozawa, Saitama 359-1192, Japan; ^6^Social Medical Corporation, The Yamatokai Foundation, 1-13-12 Nangai, Higashiyamato, Tokyo 207-0014, Japan

## Abstract

Ultraviolet (UV) irradiation induces proinflammatory responses in skin cells, including dermal fibroblasts, accelerating premature skin aging (photoaging). ETAS 50, a standardized extract from the* Asparagus officinalis* stem, is a novel and unique functional food that suppresses proinflammatory responses of hydrogen peroxide-stimulated skin fibroblasts and interleukin- (IL-) 1*β*-stimulated hepatocytes. To elucidate its antiphotoaging potencies, we examined whether ETAS 50 treatment after UV-B irradiation attenuates proinflammatory responses of normal human dermal fibroblasts (NHDFs). UV-B-irradiated NHDFs showed reduced levels of the cytosolic inhibitor of nuclear factor-*κ*B *α* (I*κ*B*α*) protein and increased levels of nuclear p65 protein. The nuclear factor-*κ*B nuclear translocation inhibitor JSH-23 abolished UV-B irradiation-induced IL-1*β* mRNA expression, indicating that p65 regulates transcriptional induction. ETAS 50 also markedly suppressed UV-B irradiation-induced increases in IL-1*β* mRNA levels. Immunofluorescence analysis revealed that ETAS 50 retained p65 in the cytosol after UV-B irradiation. Western blotting also showed that ETAS 50 suppressed the UV-B irradiation-induced increases in nuclear p65 protein. Moreover, ETAS 50 clearly suppressed UV-B irradiation-induced distribution of importin-*α* protein levels in the nucleus without recovering cytosolic I*κ*B*α* protein levels. These results suggest that ETAS 50 exerts anti-inflammatory effects on UV-B-irradiated NHDFs by suppressing the nuclear import machinery of p65. Therefore, ETAS 50 may prevent photoaging by suppressing UV irradiation-induced proinflammatory responses of dermal fibroblasts.

## 1. Introduction

Repeated ultraviolet (UV) exposure results in premature skin aging, which is defined as photoaging, characterized by deep wrinkle formation on the skin. Although all types of UV (UV-A, UV-B, and UV-C) irradiation directly damage nuclear DNA in skin cells by producing pyrimidine dimers [1], UV-A and UV-B also generate reactive oxygen species (ROS), including superoxide radicals, hydrogen peroxide, hydroxyl radicals, and singlet oxygen, which cause significant cellular damage by producing oxidative DNA legions and oxidizing cellular proteins or lipids [2, 3].

UV-A and UV-B irradiation and ROS activate a variety of intracellular signaling and downstream transcription factors. Among these are nuclear factor-*κ*B (NF-*κ*B), which regulates the expression of proinflammatory mediators [4]. Indeed, exogenous hydrogen peroxide treatment evokes tyrosine phosphorylation of inhibitor of NF-*κ*B *α* (I*κ*B*α*), leading to NF-*κ*B liberation and translocation into the nucleus [5–8]. It has been shown that UV-B irradiation promotes the production of proinflammatory cytokines, inducible nitric oxide synthase (iNOS), cyclooxygenase-2, matrix metalloproteinase (MMP)-1, and MMP-3 via NF-*κ*B signaling in primary human dermal fibroblasts [9–13]. As proinflammatory responses in skin cells promote infiltration and ROS production of immune cells in skin tissues and resulting collagen breakdown accelerates photoaging, suppressing UV-induced NF-*κ*B activation is an important target for preventing photoaging.

ETAS 50 is a standardized extract from the* Asparagus officinalis* stem produced by the Amino Up Chemical Co. Ltd. (Sapporo, Japan). It has been found to be a novel and unique functional food that attenuates sleep deprivation-induced stress responses and promotes sleep in mice and humans [14, 15]. A recent study also showed that ETAS 50 supplementation reduced the feelings of dysphoria and fatigue, ameliorated the quality of sleep, and enhanced stress-load performance as well as increasing salivary secretory immunoglobulin A levels in healthy adults [16]. In addition to these anti-stress effects, ETAS 50 has antiaging potencies via its ability to ameliorate cognitive impairment in senescence-accelerated mice [17], to protect neuronal PC-12 cells from amyloid *β* peptide-induced oxidative stress and cytotoxicity [18], and to improve learning ability in young healthy rats [19].

Moreover, we recently reported that ETAS 50 attenuates hydrogen peroxide-induced MMP-9 expression by suppressing phosphorylation of c-Jun N-terminal kinase and activator protein-1 c-Jun subunit [20], as well as hydrogen peroxide-induced IL-12*α* and iNOS expressions by suppressing the nuclear translocation of NF-*κ*B p65 subunit in murine skin L929 fibroblasts without reversing the carbonylation level of proteins, an index of protein oxidation [21]. To determine the antiphotoaging potencies of ETAS 50, this study aimed to clarify whether and how ETAS 50 prevents UV-B-induced proinflammatory responses in normal human dermal fibroblasts (NHDFs).

## 2. Materials and Methods

### 2.1. Preparation of ETAS 50

In the present study, the same batch of ETAS 50 (Amino Up Chemical Co. Ltd.) was used as in previous studies [20, 21]. Methods for preparing ETAS 50 are briefly described as follows [14, 15, 22]: (1) fresh bottom parts of asparagus were boiled in water at 121°C for 45 min; (2) the boiled bottom parts of asparagus and the resulting extract were treated with cellulase and pectinase which are widely used in food industry; (3) after these enzymes were inactivated by incubation at 121°C for 20 min, the extract was centrifuged and the resultant supernatant was mixed with dextrin (Pinedex; Matsutani Chemical Industry, Hyogo, Japan) as a filler; (4) the mixture was then concentrated* in vacuo* at 105°C, sterilized at 121°C for 45 min, and then spray-dried to produce an ETAS 50 powder consisting of 52.6% ETAS 50 and 47.4% dextrin. Component analysis revealed that the ETAS 50 powder was composed of 86.5% carbohydrates, 7.1% proteins, 2.9% ash, 1.0% lipids, and 2.5% moisture. In this study, the concentrations of ETAS 50 except for dextrin are indicated.

### 2.2. Cell Culture

NHDFs from an adult donor (PromoCell, Heidelberg, Germany) were cultured in Fibroblast Growth Medium 2 (PromoCell) supplemented with 2% fetal calf serum, 1 ng/mL basic fibroblast growth factor, and 5 *μ*g/mL insulin. The cultures were maintained at 37°C in a humidified incubator containing 5% CO_2_ in air. All experiments were conducted using cells at passage 4. Prior to the initiation of experiments, NHDFs were seeded on culture dishes or multiwell plates at a density of 1.5 × 10^4^ cells/cm^2^ and cultured for 24 h. UV-B irradiation was conducted with a UVM-57 Handheld UV Lamp (6-watt, 302 nm; UVP, LLC, Upland, CA, USA), which was applied at distance of 7.5 cm from the cells. Irradiance was measured with a UV light meter UV-340C (CUSTOM, Tokyo, Japan). To test the effect of UV-B alone, the cells were washed twice with phosphate-buffered saline and irradiated with UV-B for 30 s to obtain a total dose of 20 mJ/cm^2^ under a thin layer of phosphate-buffered saline. Immediately after irradiation, the cells were cultured in complete medium for different periods. The dose is commonly utilized in* in vitro* experiments [10, 12, 23, 24] and is within the range of natural environment because it is approximately one seventieth of the average daily accumulated amount in Japan. To assess its effects, ETAS 50 or dextrin (vehicle) was directly dissolved in complete medium to produce a final concentration of 1 mg/mL, and then the supplemented medium was sterilized using a Millex 0.22-*μ*m Syringe-Driven Filter Unit (Merck Millipore, Darmstadt, Germany) [20, 21]. Immediately after UV-B irradiation, the cells were cultured in supplemented medium for different periods. As described above, ETAS 50 powder contains approximately 50% dextrin. Therefore, ETAS 50-free control cells were treated with an equivalent concentration of dextrin to subtract dextrin effects in all experiments. An inhibitor of NF-*κ*B nuclear translocation, JSH-23 (Sigma-Aldrich, St. Louis, MO, USA), was dissolved in dimethyl sulfoxide (DMSO; Wako Pure Chemical Industries, Osaka, Japan) to generate a 100-mM solution, and then this solution was added to complete medium to achieve a final concentration of 10 *μ*M. The final volume of DMSO in the medium was equivalent (0.1%) in cells treated with and without JSH-23. JSH-23 treatment was also performed immediately after UV-B irradiation.

### 2.3. Preparation of Nuclear and Cytosol Extracts

Cytosolic proteins were extracted in lysis buffer containing 10 mM HEPES–KOH (pH 7.8), 10 mM KCl, 2 mM MgCl_2_, 0.1 mM ethylenediaminetetraacetic acid (EDTA), and 0.1% Nonidet P-40 supplemented with a Complete Protease Inhibitor Cocktail Tablet (Roche Life Science, Basel, Switzerland) and Phosphatase Inhibitor Cocktail (Nacalai Tesque, Kyoto, Japan). After low-speed centrifugation (200 ×*g*) at 4°C for 5 min, sediments containing nuclei were resuspended in wash buffer containing 250 mM sucrose, 10 mM HEPES–KOH (pH 7.8), 10 mM KCl, 2 mM MgCl_2_, and 0.1 mM EDTA supplemented with inhibitor cocktails. After low-speed centrifugation (200 ×*g*) at 4°C for 5 min, the sediments were resuspended in nuclear extraction buffer containing 50 mM HEPES–KOH (pH 7.8), 420 mM KCl, 5 mM MgCl_2_, 0.1 mM EDTA, and 20% glycerol supplemented with inhibitor cocktails and rotated at 4°C for 30 min. After centrifugation at 13,000 ×*g* at 4°C for 15 min, the supernatants were used as nuclear proteins. The concentrations of cytosolic and nuclear proteins were determined by using a BCA Protein Assay Kit (Pierce Biotechnology, Rockford, IL, USA).

### 2.4. Western Blotting

Protein samples (10 *μ*g) were denatured by incubation at 95°C for 5 min in sample buffer containing 75 mM Tris-HCl (pH 6.8), 0.6% sodium dodecyl sulfate, 15% glycerol, 7.5%  *β*-mercaptoethanol, and 9 *μ*g/mL bromophenol blue. The denatured proteins were separated by electrophoresis on a sodium dodecyl sulfate-polyacrylamide gel and then transferred to a polyvinylidene difluoride membrane (GE Healthcare, Little Chalfont, UK). Each membrane was blocked with 5% bovine serum albumin, and primary antibodies against p65, lamin A/C, I*κ*B*α*, GAPDH (Cell Signaling Technology, Danvers, MA, USA), or importin-*α* (Sigma-Aldrich) were applied at a 1/1,000 dilution at 4°C overnight. Secondary antibody (horseradish peroxidase-conjugated AffiniPure Mouse Anti-Rabbit IgG or Goat Anti-Mouse IgG; Jackson ImmunoResearch Laboratories, West Grove, PA, USA) was applied at a 1/20,000 dilution for 30 min. The membrane was incubated with Clarity Western ECL Substrate (Bio-Rad Laboratories, Hercules, CA, USA) and exposed to X-ray film. The density of each protein band was quantified using ImageJ software (National Institutes of Health, Bethesda, MD, USA). Lamin A/C and GAPDH are commonly utilized as a marker and loading control for the nuclear and cytosolic protein fractions, respectively, and UV-B irradiation did not significantly change their band density. Therefore, the expression levels of target proteins in the nucleus and cytosol were calculated as the ratios of their values to those of lamin C and GAPDH as internal controls, respectively.

### 2.5. Reverse Transcription and Real-Time Polymerase Chain Reaction (PCR)

Total cellular RNA was extracted using RNAiso Plus reagent (TaKaRa Bio, Shiga, Japan). One microgram of total cellular RNA was converted to single-stranded cDNA using the PrimeScript 1st Strand cDNA Synthesis Kit (Takara Bio). cDNA (1 *μ*L) was amplified in a FastStart Universal Probe Master (Roche Life Science) using the 7500 Real-Time PCR System (Thermo Fisher Scientific, Waltham, MA, USA). The PCR conditions were 50°C for 2 min and 95°C for 15 s, followed by 45 cycles at 95°C for 15 s and 60°C for 1 min. The primers and fluorescent probes were as follows: IL-1*β*, forward 5′-TAC CTG TCC TGC GTG TTG AA-3′, reverse 5′-TCT TTG GGT AAT TTT TGG GAT CT-3′, Universal Probe #76 (Roche Life Science); 18S, forward 5′-AAA TCA GTT ATG GTT CCT TTG GTC-3′, reverse 5′-GCT CTA GAA TTA CCA CAG TTA TCC AA-3′, Universal Probe #55 (Roche Life Science). The expression level of IL-1*β* mRNA was calculated as the ratio of its value to that of 18S rRNA as an internal control. The amplification charts of IL-1*β* and 18S obtained from each experiment are shown in Supplementary Figure 1.

### 2.6. Fluorescence Immunomicroscopy

The cells were fixed with 4% paraformaldehyde for 15 min and then permeabilized with methanol at −20°C for 10 min. After blocking with 4% bovine serum albumin, the primary antibody against p65 (Cell Signaling) was applied at a 1/400 dilution for 1 h. Secondary antibody (Goat Anti-Rabbit IgG H&L [Alexa Fluor 488]; Abcam, Cambridge, UK) was applied at a 1/1,000 dilution for 30 min with 10 *μ*g/mL propidium iodide (Sigma-Aldrich). After mounting with Fluoro-KEEPER Antifade Reagent (Nacalai Tesque), subcellular localization of p65 and nucleus was visualized using a Nikon BioStation IM (NIKON, Tokyo, Japan) with FL1 and FL2 detectors, respectively. The ratio of nuclear p65-positive cells to total cells in the fields was calculated.

### 2.7. Statistical Analysis

Experimental data are presented as the mean ± standard error of the mean (SEM). Differences between two groups were assessed using Student's* t*-test. Comparisons among at least three groups were tested by one-way analysis of variance (ANOVA), and then post hoc comparisons to determine significant differences between groups were performed using Tukey's test. Differences were considered statistically significant when* p* values were less than 0.05.

## 3. Results and Discussion

**Figure 1:**
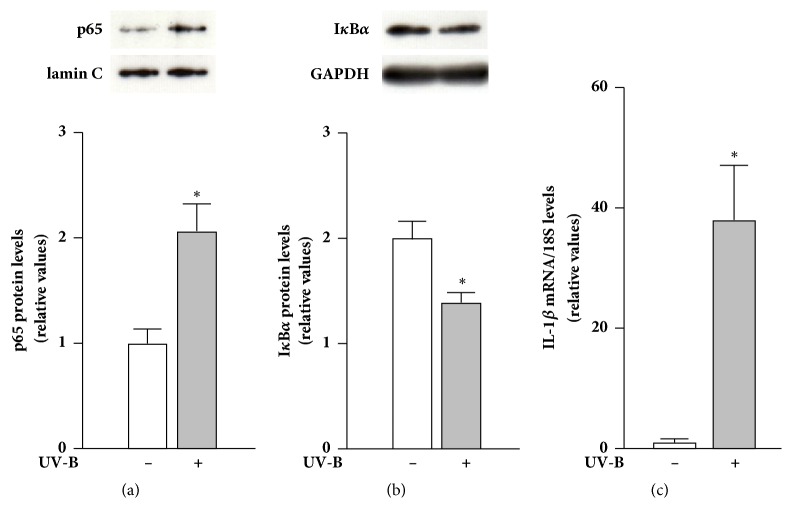
Proinflammatory responses of NHDFs irradiated with UV-B. (a) Effect of UV-B irradiation on the amount of p65 protein in the nuclear fraction of the cells. (b) Effect of UV-B irradiation on the amount of I*κ*B*α* protein in the cytosolic fraction of the cells. The cells were cultured for 3 h after UV-B irradiation (20 mJ/cm^2^). p65 and I*κ*B*α* were detected by western blotting. Data are the relative ratios of p65 to lamin C and of I*κ*B*α* to GAPDH. (c) Effect of UV-B irradiation on the level of IL-1*β* mRNA of the cells. The cells were cultured for 24 h after UV-B irradiation (20 mJ/cm^2^). IL-1*β* mRNA levels were analyzed by real-time PCR. The relative ratio of IL-1*β* to 18S is shown. Means ± SEM (*n* = 3). *∗p* < 0.05 (by Student's* t*-test).

Several studies have shown that UV-B irradiation triggers nuclear translocation of NF-*κ*B in skin cells, including primary human dermal fibroblasts, the HaCaT keratinocyte cell line, and murine skin tissues, in* in vitro* and* in vivo* experimental models [9, 13, 23, 24]. According to these observations, we first confirmed whether p65 was translocated into the nucleus in response to UV-B irradiation in NHDFs. The amount of p65 protein in the nuclear fraction of cells irradiated with UV-B was significantly higher than that in nonirradiated cells after 3 h of culture (Figure 1(a)). After 3 h, the amount of I*κ*B*α* protein in the cytosolic fraction of cells irradiated with UV-B was significantly lower than that in nonirradiated cells (Figure 1(b)). These results suggest that UV-B irradiation induces p65 nuclear translocation accompanied by I*κ*B*α* degradation in NHDFs.

**Figure 2:**
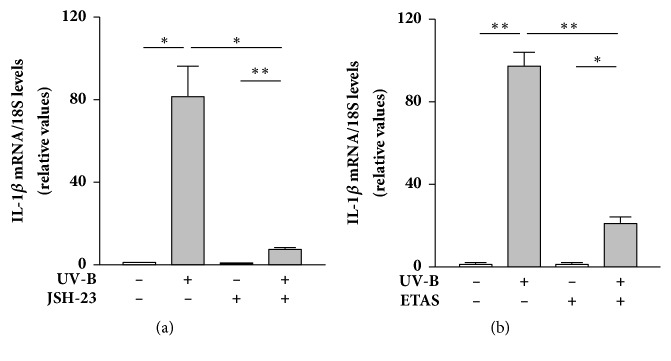
Effect of NF-*κ*B inhibitor JSH-23 or ETAS on UV-B irradiation-induced IL-1*β* mRNA expression in NHDFs. (a) Effect of JSH-23 on UV-B-induced IL-1*β* mRNA expression in the cells. The cells were treated with 10 *μ*M JSH-23 or DMSO alone for 24 h after UV-B irradiation (20 mJ/cm^2^). (b) Effect of ETAS on UV-B-induced IL-1*β* mRNA expression in the cells. The cells were treated with 1 mg/mL ETAS or dextrin for 24 h after UV-B irradiation (20 mJ/cm^2^). IL-1*β* mRNA levels were analyzed by real-time PCR. The relative ratios of IL-1*β* to 18S are shown. Means ± SEM (*n* = 3). *∗p* < 0.05, *∗∗p* < 0.01 (by one-way ANOVA and Tukey's test).

Inflammatory process in skin tissues after UV irradiation largely contributes to premature skin aging [25]. The main source of proinflammatory cytokines, such as IL-1, IL-6, and tumor necrosis factor-*α*, in UV-B-irradiated skin tissue is keratinocytes in the epidermis, and these cytokines indirectly activate dermal fibroblasts to produce MMPs, elastase, and other enzymes, leading to collagen and elastin breakdown [26–28]. UV-A and UV-B also directly activate dermal fibroblasts to induce the transcription of proinflammatory cytokines [10, 29, 30]. Indeed, we found that NHDFs irradiated with UV-B contained dramatically higher amounts of IL-1*β* mRNA than nonirradiated cells (Figure 1(c)). To evaluate whether NF-*κ*B mediates the transcriptional induction of IL-1*β*, NHDFs were treated with the NF-*κ*B nuclear translocation inhibitor JSH-23 after UV-B irradiation. JSH-23 treatment after UV-B irradiation dramatically suppressed the increased level of IL-1*β* mRNA (Figure 2(a)), indicating that p65 translocated into the nucleus after UV-B irradiation regulates IL-1*β* mRNA expression in NHDFs.

**Figure 3:**
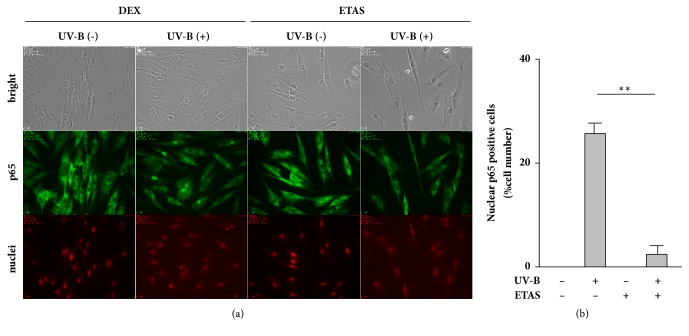
Effect of ETAS on UV-B irradiation-induced p65 subcellular localization change in NHDFs. The cells were treated with 1 mg/mL ETAS or dextrin for 1 h after UV-B irradiation (20 mJ/cm^2^). (a) p65 and nuclei were probed with Alexa Fluor 488 and propidium iodide, which were visualized using FL1 (green fluorescence) and FL2 (red fluorescence) detectors, respectively, at 20× magnification. Data shown are representative of similar results from three independent experiments. (b) The ratio of nuclear p65-positive cells to total cells in the fields was calculated. Means ± SEM (*n* = 6). *∗∗p* < 0.01 (by one-way ANOVA and Tukey's test).

Many* in vitro* and* in vivo* studies have reported that extracts or certain bioactive compounds derived from a wide variety of plants exert antiphotoaging effects by suppressing NF-*κ*B activation and resulting proinflammatory responses in skin cells after UV irradiation [9–12, 23, 24, 30, 31]. In this context, we examined whether ETAS 50 suppresses UV-B irradiation-induced proinflammatory signaling in NHDFs. We found that ETAS 50 treatment after UV-B irradiation significantly suppressed the increased IL-1*β* mRNA levels in cells as JSH-23 treatment did (Figure 2(b)). Moreover, fluorescence immunomicroscopy revealed that ETAS 50 treatment retained p65 protein in the cytosol and suppressed the increased number of nuclear p65-positive cells after UV-B irradiation (Figure 3). These results indicate that ETAS 50 suppresses IL-1*β* mRNA production by repressing NF-*κ*B nuclear translocation in UV-B-irradiated NHDFs.

Proinflammatory responses in skin cells promote infiltration and ROS production of immune cells in skin tissues and resulting collagen breakdown accelerates photoaging. Indeed, IL-1*β* triggers gene expression of certain types of MMPs in keratinocytes [32, 33]. Among the MMPs, MMP-9 catalyzes degradation of type IV collagen, which comprises the basement membrane of the dermoepidermal junction [34]. In addition, MMP-1 catalyzes degradation of type I collagen in concert with MMP-9, which comprises a large amount of dermal collagen [25]. The breakdown of type IV and type I collagen in the dermis causes deep wrinkle formation. Therefore, ETAS 50 has potentials to prevent gene expression of MMPs and preserve the quantity of collagen by its anti-inflammatory effects.

**Figure 4:**
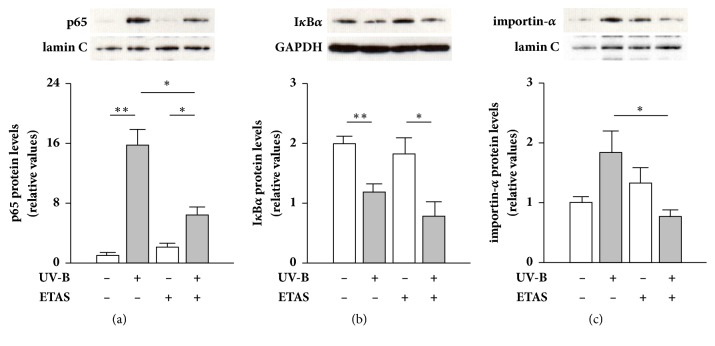
Effect of ETAS on UV-B irradiation-induced activation of p65 nuclear translocation machinery in NHDFs. (a) Effect of ETAS on UV-B-induced nuclear p65 accumulation in the cells. (b) Effect of ETAS on UV-B-induced cytosolic I*κ*B*α* degradation in the cells. (c) Effect of ETAS on UV-B-induced distribution of importin-*α* in the nucleus of cells. The cells were treated with 1 mg/mL ETAS or dextrin for 3 h after UV-B irradiation (20 mJ/cm^2^). p65, I*κ*B*α*, and importin-*α* were detected by western blotting. The relative ratios of p65 or importin-*α* to lamin C and of I*κ*B*α* to GAPDH are shown. Means ± SEM (*n* = 4). *∗p* < 0.05, *∗∗p* < 0.01 (by one-way ANOVA and Tukey's test).

Suppressive effects of plant extracts and phytochemicals on NF-*κ*B activity have been commonly explained by their antioxidant behaviors [9, 10, 30], but a few studies have examined these effects. In contrast, a study showed that PPAR *α*/*γ* dual agonist improved photoaging characteristics on UV-B-irradiated murine skin by suppressing NF-*κ*B nuclear accumulation and the resulting cytotoxic peroxynitrite production without scavenging ROS [13]. Therefore, we next explored how ETAS 50 repressed UV-B irradiation-induced p65 nuclear translocation. Although ETAS 50 significantly repressed the accumulation of p65 protein in the nuclear fraction after UV-B irradiation (Figure 4(a)), ETAS 50 treatment did not influence the reduced level of cytosolic I*κ*B*α* protein after UV-B irradiation (Figure 4(b)), suggesting that ETAS 50 inhibits nuclear translocation of p65 without affecting the machinery involved in its liberation from I*κ*B*α*. We also recently reported that ETAS 50 pretreatment attenuated hydrogen peroxide-induced IL-12*α* and iNOS mRNA expression by suppressing p65 nuclear accumulation without restoring cytosolic I*κ*B*α* protein levels in murine L929 skin fibroblast cells [21], and ETAS 50 did not reduce hydrogen peroxide-induced protein carbonylation, an index of protein oxidation [20]. The present and previous findings suggest that ETAS 50 modulates the machinery for p65 nuclear import without affecting upstream events after UV irradiation, such as ROS generation and ROS-induced signal transduction, which mediates p65 liberation from I*κ*B*α*.

The import of transcription factors from the cytosol to the nucleus is regulated by the importin-*α* and -*β* family of proteins [35]. Importin-*α*s are adaptor proteins that recruit importin-*β* via binding to the nuclear localization signal domain of cargo proteins, and the heterotrimeric complex is imported into the nucleus through the nuclear pore complex [35]. It has been reported that nuclear import of p65 is mediated by importin-*α*1, -*α*3, -*α*5, and -*α*6 [36–38]. In this study, we found that NHDFs treated with ETAS 50 after UV-B irradiation had lower amounts of importin-*α* protein in the nuclear fraction than in nontreated cells (Figure 4(c)). These results suggest that ETAS 50 suppresses UV-B irradiation-stimulated activation of the nuclear import system mediated by importin-*α*.

Another group showed that ETAS 50 contains 5-hydroxymethyl-2-furfural and its unique derivative asfural, which is not present in raw asparagus [22]; these compounds exert anti-inflammatory effects on IL-1*β*-induced iNOS production in rat primary hepatocytes [39]. The previous study showed that the anti-inflammatory effects of ETAS 50 and its compounds were mediated by their ability to induce heat shock protein 70 production [39]. However, we did not observe these effects in this study (unpublished data). Although other bioactive compounds have not been identified in ETAS 50, it is possible that unidentified compounds mediate the unique effects on UV-irradiated dermal fibroblasts.

## 4. Conclusion

ETAS 50 shows potential for preventing skin aging through anti-inflammatory effects mediated by its capability to suppress NF-*κ*B nuclear import machinery during UV-B irradiation. Therefore, this readily available, inexpensive, and eco-friendly functional food may be a useful component in dermatologic prophylactic strategies for maintaining skin health and function.

## Data Availability

The data used to support the findings of this study are available from the corresponding author upon request.

## Supplementary Materials

Supplementary Materials Supplementary Figure 1: The amplification chart of IL-1*β* and 18S obtained from each experiment by real-time PCR. (a) Effect of UV-B irradiation on the level of IL-1*β* mRNA of NHDFs (see Figure 1(c)). (1) the UV-B (-) group and (2) the UV-B (+) group. (b) Effect of JSH-23 on UV-B-induced IL-1*β* mRNA expression in NHDFs (see Figure 2(a)). (1) the UV-B (+)/JSH-23 (-) group; (2) the UV-B (+)/JSH-23 (+) group; (3) the UV-B (-)/JSH-23 (-) group; and (4) the UV-B (-)/JSH-23 (+) group. (c) Effect of ETAS on UV-B-induced IL-1*β* mRNA expression in NHDFs (see Figure 2(b)). (1) the UV-B (+)/ETAS (-) group; (2) the UV-B (+)/ETAS (+) group; and (3) the UV-B (-)/ETAS (-) and UV-B (-)/ETAS (+) groups. Since the present study used TaqMan probes for cDNA amplification, melt peak analysis could not be done.
