# Supplementary material for: Anti-Inflammatory Effect of ETAS®50 by Inhibiting Nuclear Factor-κB p65 Nuclear Import in Ultraviolet-B-Irradiated Normal Human Dermal Fibroblasts
Source: Evid Based Complement Alternat Med. 2018 Jun 3;2018:5072986. doi: 10.1155/2018/5072986 (PMC6008667; doi:10.1155/2018/5072986)
Supplement: Supplementary Materials — Supplementary Figure 1: The amplification chart of IL-1β and 18S obtained from each experiment by real-time PCR. (a) Effect of UV-B irradiation on the level of IL-1β mRNA of NHDFs (see Figure 1(c)). (1) the UV-B (-) group and (2) the UV-B (+) group. (b) Effect of JSH-23 on UV-B-induced IL-1β mRNA expression in NHDFs (see Figure 2(a)). (1) the UV-B (+)/JSH-23 (-) group; (2) the UV-B (+)/JSH-23 (+) group; (3) the UV-B (-)/JSH-23 (-) group; and (4) the UV-B (-)/JSH-23 (+) group. (c) Effect of ETAS on UV-B-induced IL-1β mRNA expression in NHDFs (see Figure 2(b)). (1) the UV-B (+)/ETAS (-) group; (2) the UV-B (+)/ETAS (+) group; and (3) the UV-B (-)/ETAS (-) and UV-B (-)/ETAS (+) groups. Since the present study used TaqMan probes for cDNA amplification, melt peak analysis could not be done. [file 5072986.f1.pdf]

Evidence-Based Complementary and Alternative Medicine

Research Article

Anti-Inflammatory Effect of ETAS®50 by Inhibiting Nuclear Factor-κB p65 Nuclear Import in Ultraviolet-B-Irradiated Normal Human Dermal Fibroblasts

Ken Shirato,[1] Tomoko Koda,[2] Jun Takanari,[3] Takuya Sakurai,[1] Junetsu Ogasawara,[4] Kazuhiko Imaizumi,[5] Hideki Ohno,[6] and Takako Kizaki[1]

[1]Department of Molecular Predictive Medicine and Sport Science, Kyorin University School of Medicine, 6-20-2 Shinkawa, Mitaka, Tokyo 181-8611, Japan

[2]Faculty of Nursing, Tokyo Healthcare University, 2-5-1 Higashigaoka, Meguro, Tokyo 152-8558, Japan

[3]Amino Up Chemical Co. Ltd., 363-32 Shin-ei, Kiyota, Sapporo, Hokkaido 004-0839, Japan

[4]Department of Health Science, Asahikawa Medical University, 2-1-1-1 Midorigaoka-Higashi, Asahikawa, Hokkaido 078-8510, Japan

[5]Faculty of Human Sciences, Waseda University, 2-579-15 Mikajima, Tokorozawa, Saitama 359-1192, Japan

[6]Social Medical Corporation, the Yamatokai Foundation, 1-13-12 Nangai, Higashiyamato, Tokyo 207-0014, Japan

Correspondence should be addressed to Ken Shirato; shirato@ks.kyorin-u.ac.jp

(a)

IL-1β

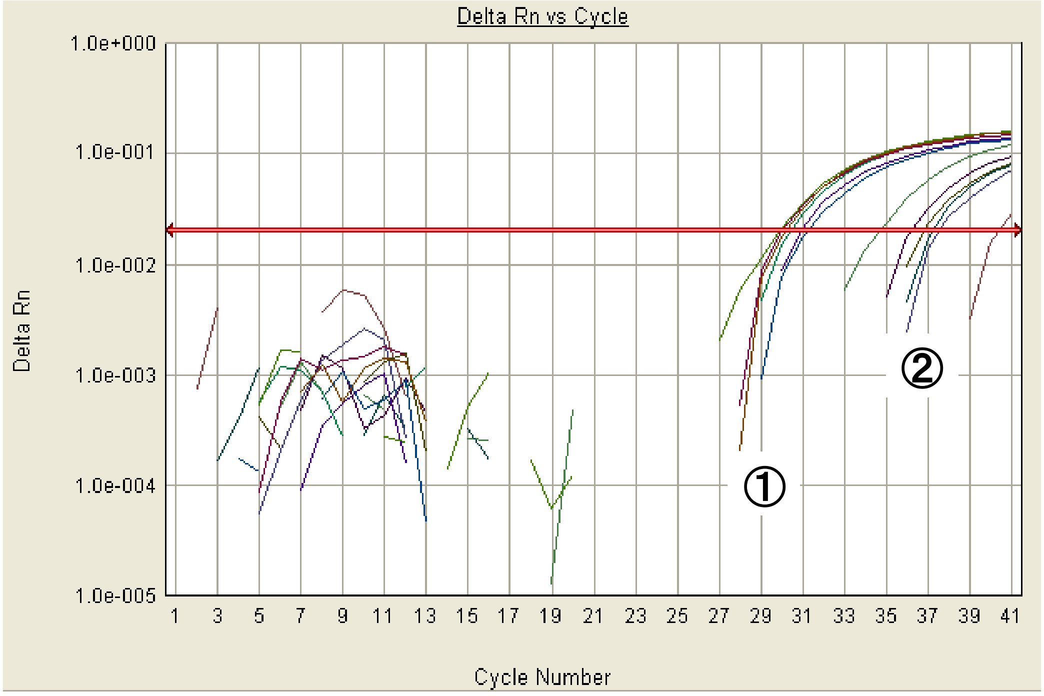

Delta Rn vs Cycle
1.0e+000
1.0e-001
1.0e-002
1.0e-003
1.0e-004
1.0e-005
Delta Rn
1 3 5 7 9 11 13 15 17 19 21 23 25 27 29 31 33 35 37 39 41
Cycle Number
①
②

18S

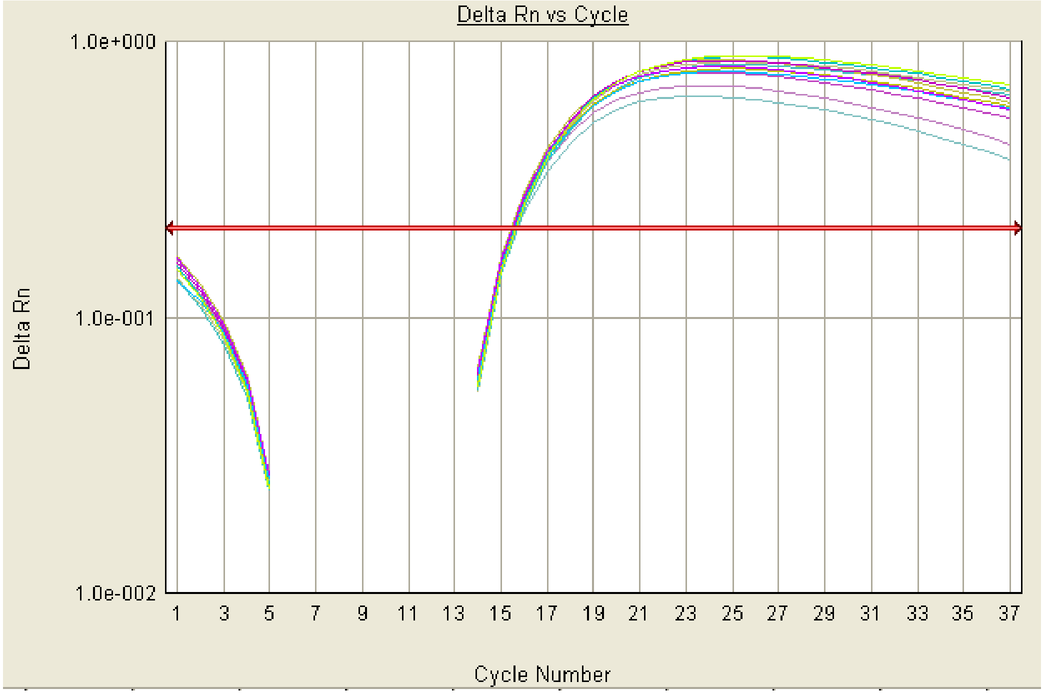

Delta Rn vs Cycle
1.0e+000
1.0e-001
1.0e-002
Delta Rn
1 3 5 7 9 11 13 15 17 19 21 23 25 27 29 31 33 35 37
Cycle Number

(b)

IL-1β

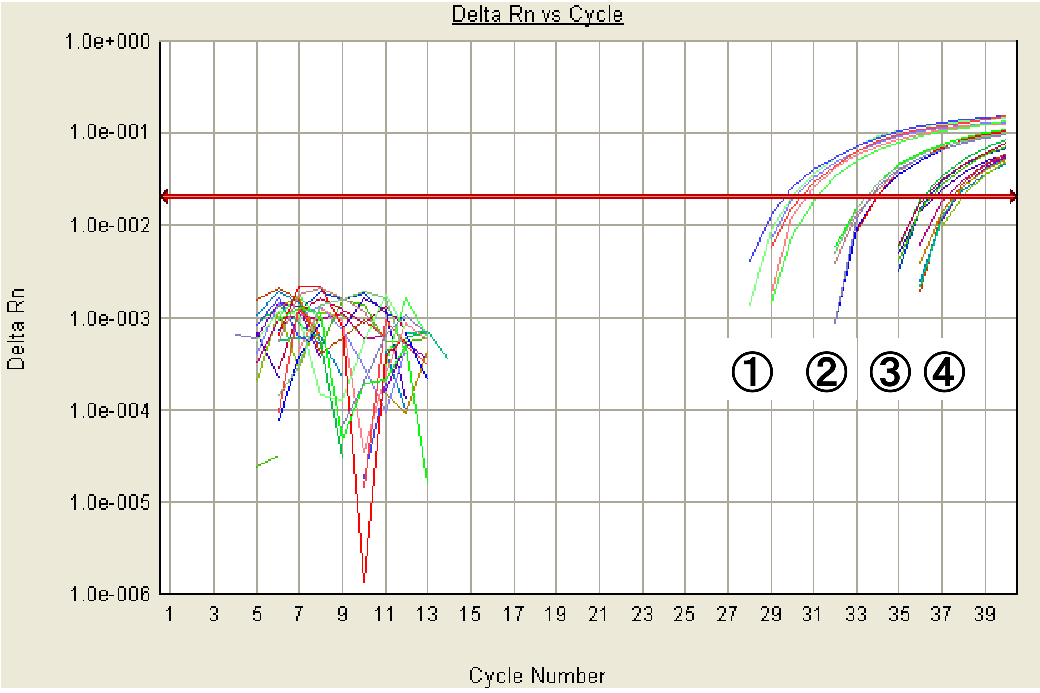
Delta Rn vs Cycle
1.0e+000
1.0e-001
1.0e-002
1.0e-003
1.0e-004
1.0e-005
1.0e-006
Delta Rn
① ② ③ ④
1 3 5 7 9 11 13 15 17 19 21 23 25 27 29 31 33 35 37 39
Cycle Number

18S

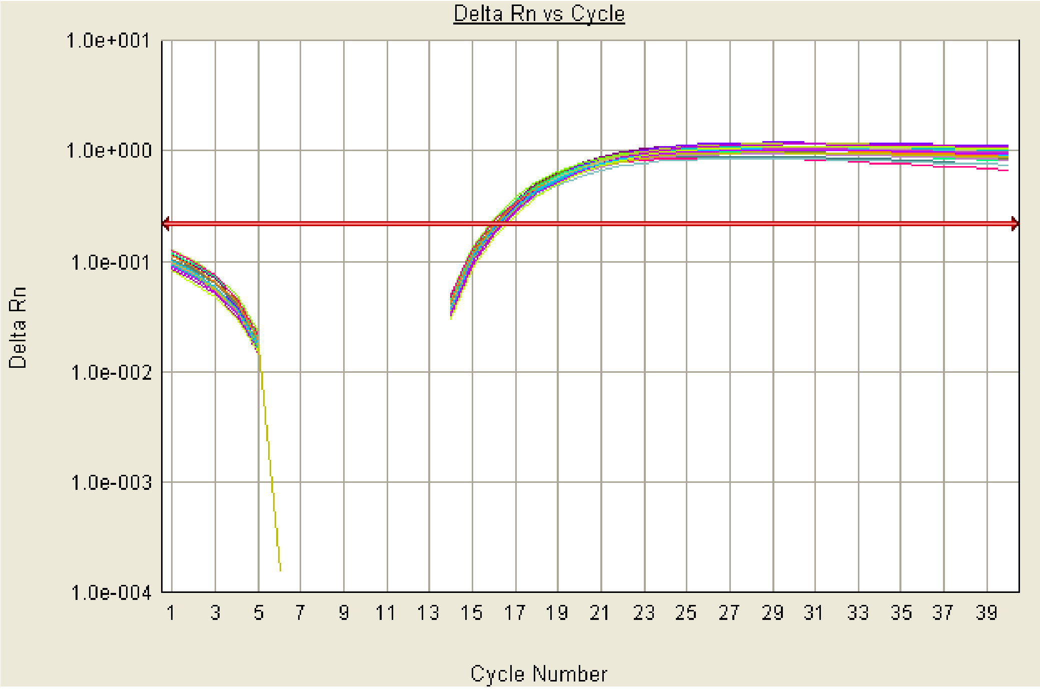
Delta Rn vs Cycle
1.0e+001
1.0e+000
1.0e-001
1.0e-002
1.0e-003
1.0e-004
Delta Rn
1 3 5 7 9 11 13 15 17 19 21 23 25 27 29 31 33 35 37 39
Cycle Number

(c)

IL-1β

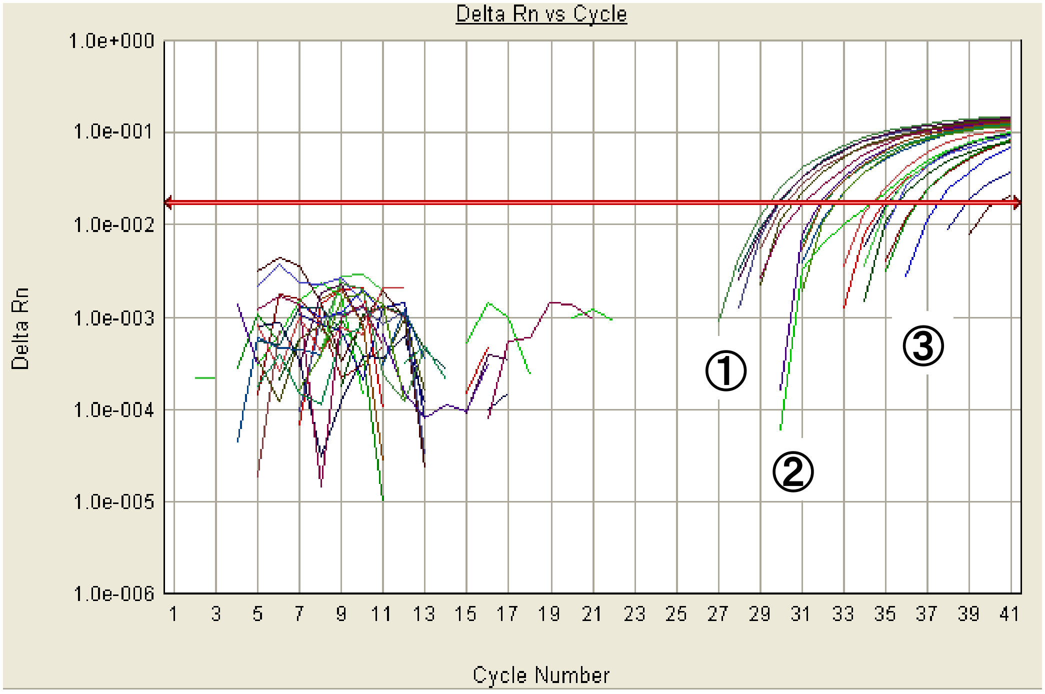
Delta Rn vs Cycle
1.0e+000
1.0e-001
1.0e-002
1.0e-003
1.0e-004
1.0e-005
1.0e-006
Delta Rn
1 3 5 7 9 11 13 15 17 19 21 23 25 27 29 31 33 35 37 39 41
Cycle Number
①
②
③

18S

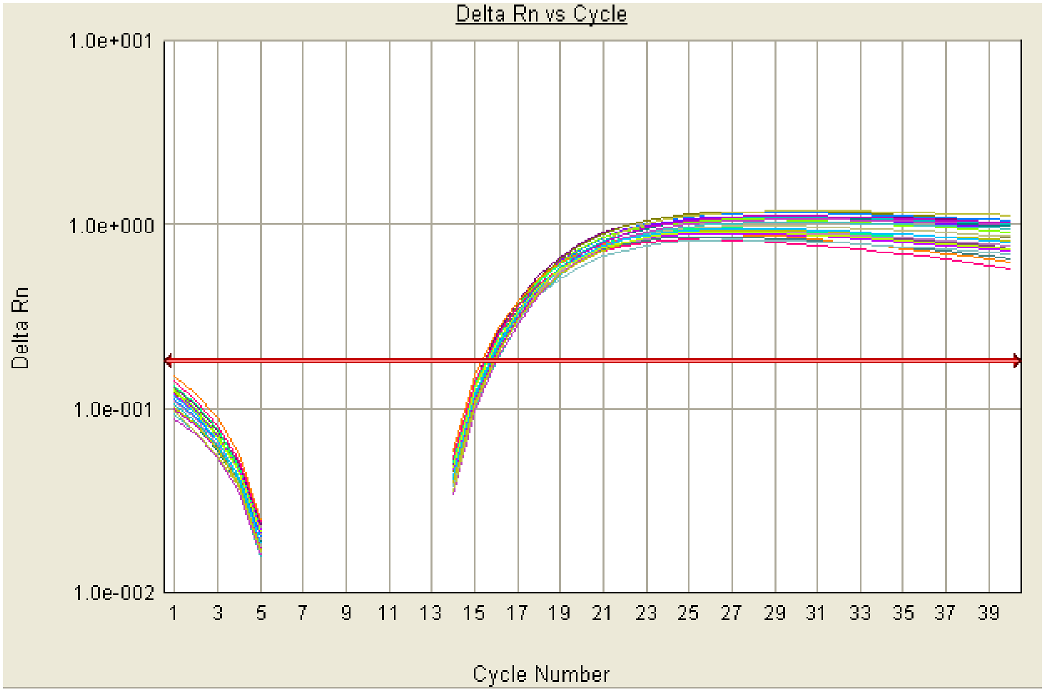
Delta Rn vs Cycle
1.0e+001
1.0e+000
1.0e-001
1.0e-002
Delta Rn
1 3 5 7 9 11 13 15 17 19 21 23 25 27 29 31 33 35 37 39
Cycle Number

Supplementary FIGURE 1: The amplification chart of IL-1β and 18S obtained from each experiment by real-time PCR. (a) Effect of UV-B irradiation on the level of IL-1β mRNA of NHDFs (see Figure 1(c)). (1) the UV-B (-) group and (2) the UV-B (+) group. (b) Effect of JSH-23 on UV-B-induced IL-1β mRNA expression in NHDFs (see Figure 2(a)). (1) the UV-B (+)/JSH-23 (-) group; (2) the UV-B (+)/JSH-23 (+) group; (3) the UV-B (-)/JSH-23 (-) group; and (4) the UV-B (-)/JSH-23 (+) group. (c) Effect of ETAS on UV-B-induced IL-1β mRNA expression in NHDFs (see Figure 2(b)). (1) the UV-B (+)/ETAS (-) group; (2) the UV-B (+)/ETAS (+) group; and (3) the UV-B (-)/ETAS (-) and UV-B (-)/ETAS (+) groups. Since the present study used TaqMan probes for cDNA amplification, melt peak analysis could not be done.
